# Reliable Neuronal Systems: The Importance of Heterogeneity

**DOI:** 10.1371/journal.pone.0080694

**Published:** 2013-12-04

**Authors:** Johannes Lengler, Florian Jug, Angelika Steger

**Affiliations:** 1 Institute of Theoretical Computer Science, ETH Zürich, Zürich, Switzerland; 2 Collegium Helveticum, Zürich, Switzerland; 3 Max-Planck Institute of Molecular Cell Biology and Genetics, Dresden, Germany; McGill University, Canada

## Abstract

For every engineer it goes without saying: in order to build a reliable system we need components that consistently behave precisely as they should. It is also well known that neurons, the building blocks of brains, do not satisfy this constraint. Even neurons of the same type come with huge variances in their properties and these properties also vary over time. Synapses, the connections between neurons, are highly unreliable in forwarding signals. In this paper we argue that both these fact add variance to neuronal processes, and that this variance is not a handicap of neural systems, but that instead predictable and reliable functional behavior of neural systems depends crucially on this variability. In particular, we show that higher variance allows a recurrently connected neural population to react more sensitively to incoming signals, and processes them faster and more energy efficient. This, for example, challenges the general assumption that the intrinsic variability of neurons in the brain is a defect that has to be overcome by synaptic plasticity in the process of learning.

## Introduction

**Figure 1:**
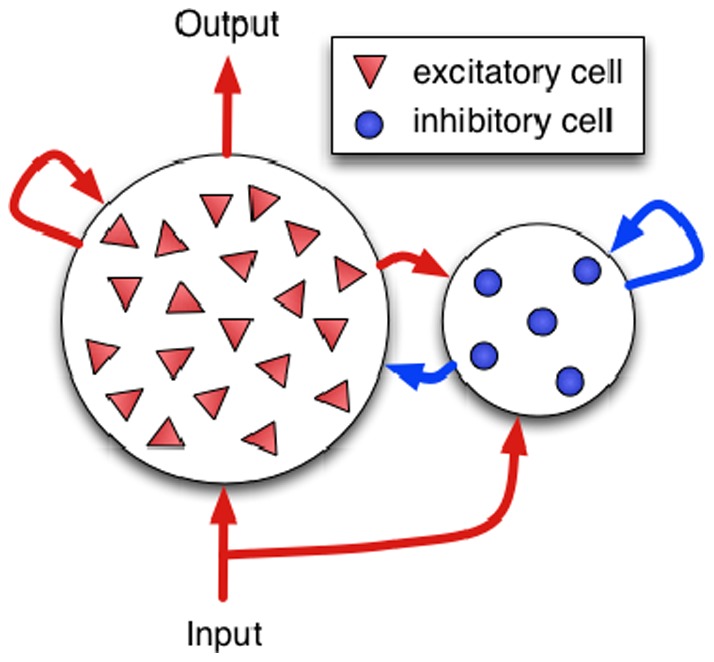
Network. Schematic representation of our recurrent network (cf. text).

A main difference between computers and the human brain is that computers are composed of extremely reliable components with failure rates as small as 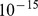 [1], while the failure rate of vesicle release at a synaptic site is 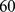
–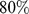 [2], meaning that failure of vesicle release is rather the rule than an exception. Another difference is that computers contain billions of identical gates, while neurons in the brain are highly individual [3], [4]. These seemingly different aspects of the brain have a joint effect: both add variance to signal processing. At first glance, this appears like an hindrance for neuronal networks to be reliable. However, in other contexts variance has proven to enhance the inherent information in a system. In particular, it has been shown that extrinsic noise can lead to a more reliable and efficient signal processing in the crayfish [5] and other animals [6], [7]. This effect is known as “stochastic resonance” and is particularly well-established in the theory of coupled oscillators [8]. In this article we demonstrate a similar, but *intrinsic* mechanism for neuronal networks. Our simulations show that the heterogeneity of neurons and unreliability of synaptic transmission increase speed, responsiveness, and even robustness of networks of spiking neurons as depicted in Figure 1.

**Figure 2:**
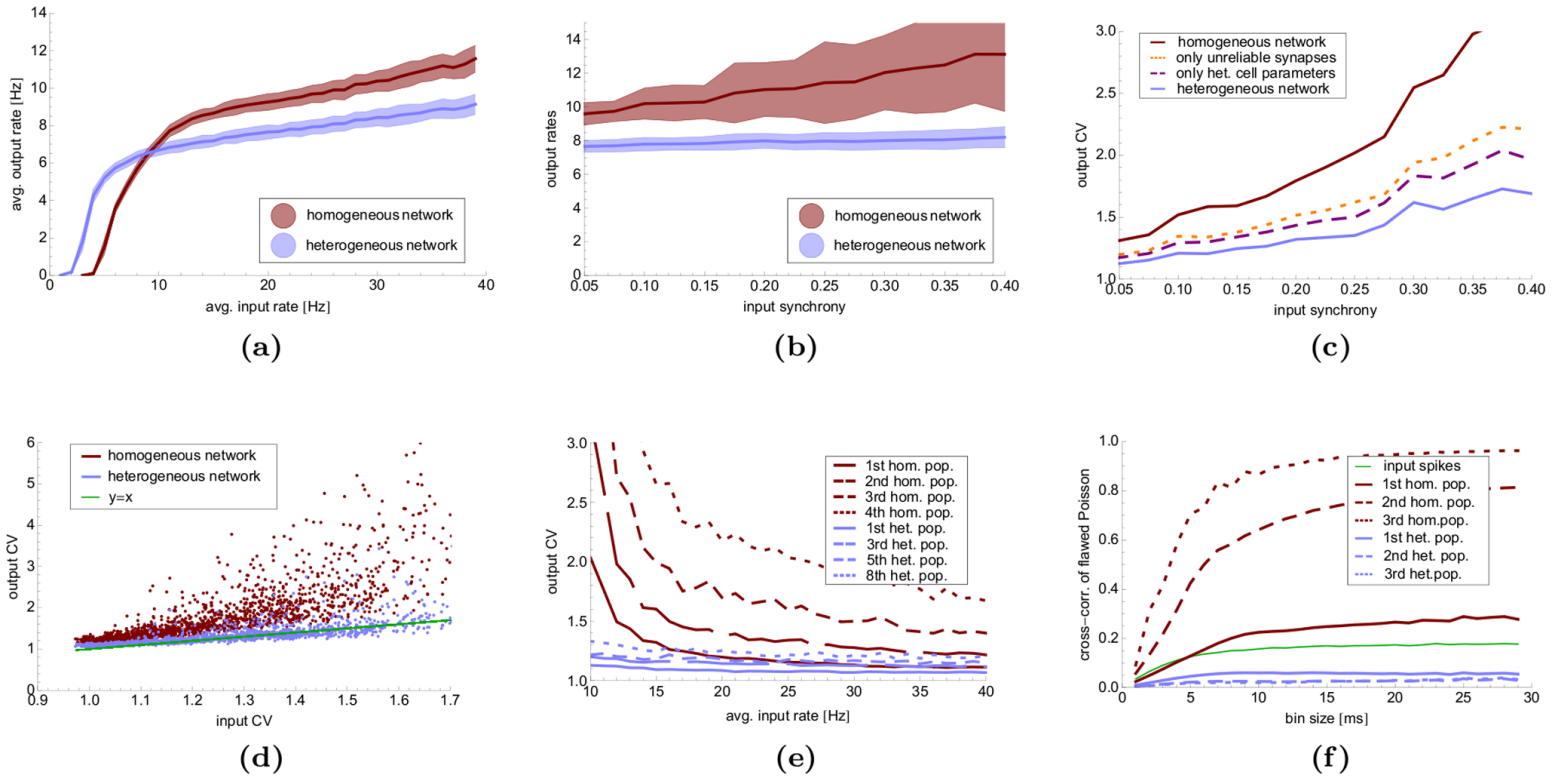
Response to Poisson Input. (**a**) Input-response curve of a heterogeneous (blue) and homogeneous (red) population for pure Poisson input (250 neurons) of varying rates. The shaded areas shows the standard deviation over 100 trials, each lasting 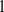 s. The synaptic weights are chosen in a way such that the network has a high dynamic range. (**b–d**) Behavior of the network in response to flawed Poisson input of 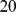 Hz; 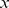-axis measures the synchronization (cf. text). (**b**) The output rate in the heterogeneous network (blue) remains unaffected while the homogenous network reacts with an increase of the output rate *and* an increasing variance; shaded areas show the standard deviation over 100 trials. (**c**) The reason for the behavior in (**b**): the coefficient of variation (CV) of the interspike times increases only very slowly in the heterogenous case (blue curve), but quickly moves to high values in the homogeneous case (red curve); high CV values of the heterogeneous network are not caused by a single parameter: eliminating variance only from neuronal properties (purple) or by just making synapse 100% reliable (brown) increase the CV values only slightly above the blue curve. (Curves show mean values of the experiments in (**b**)). (**d**) Data points from experiments in (**b**); 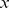-axis corresponds to CV-value of input. As input is identical to homogeneous and heterogenous networks, each input gives rise to a blue point (heterogeneous network) and to a red point (homogeneous network) at the same 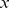-value; the plot shows that the heterogeneous network has strictly smaller CV values. (**e,f**): Behavior if the output of the network is fed as input to an additional network; here we study the effect on a sequence of up to eight such populations. (**e**): Coefficient of variation in various population for Poisson input with a given rate, blue: heterogeneous network, red: homogeneous network, green: input; curves show means of 20 trials. Note that we show population 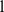,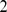,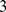, and 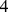 for homogeneous the network, and population 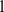, 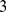, 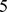, and 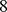 for the heterogeneous network (**f**) Cross correlation for flawed Poisson input (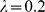) as a function of the bin size (in ms); curves show means of the cross-variances of 20 experiments, each using 20 trials to compute the cross-variance.

**Figure 3:**
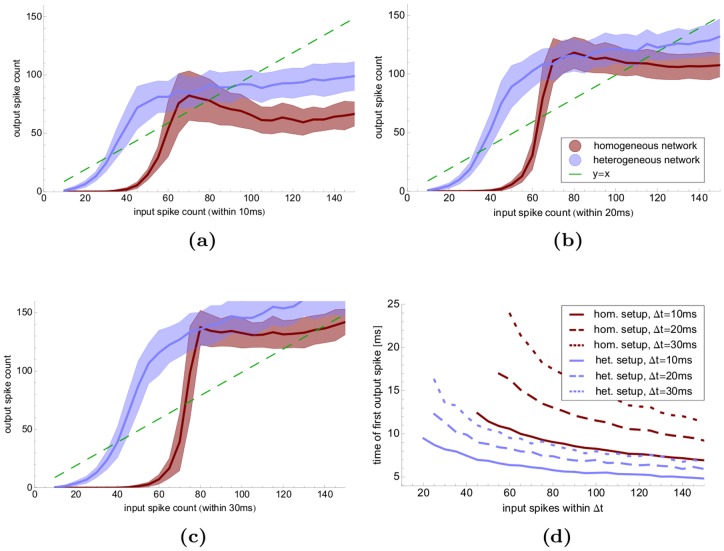
Response to Flanks. Behavior of a heterogeneous (blue) and homogeneous (red) population in response to a single input flank. (**a–c**) 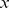-axes denote the number of input neurons that spike, 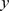-axes the number of neurons that spike within the population; input spikes are randomly distributed within an interval of (**a**) 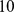 ms, (**b**) 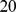 ms, (**c**) 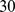 ms, shaded regions show standard deviation of 100 trials. Note that a broader input distribution leads to more spikes – at the price of a later activation of the population: (**d**) shows the time of the first spike in the population as a function of the number of input neurons (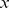-axis) and size of the input interval: 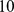 ms: solid lines, 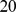 ms: dashed lines, 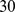 ms: dotted lines, blue: heterogeneous network, red: homogeneous network. The curves start at the input size where all 100 trials produced at least one spike. The heterogeneous network can be activated by fewer input spikes, and reacts faster.

**Figure 4:**
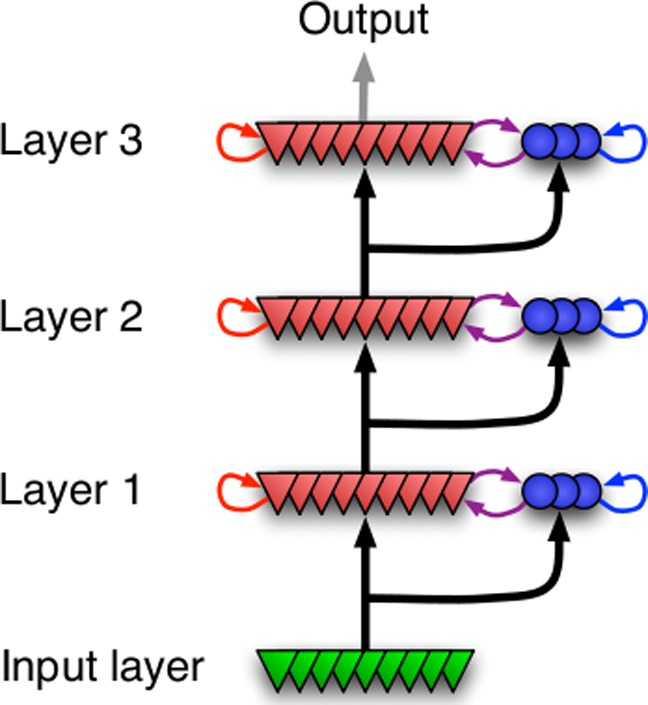
Feed Forward Model. A feed-forward chain of several populations (here for 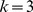), as used in Fig. 2 (e,f).

Over the last decades a lot of theoretical work has been invested in understanding neuronal coding and signal processing [9], [10]. Most of these investigations studied neural behavior in abstract networks consisting of highly simplified and identical neurons. This approach is based on a principle that proved very successful in mathematics and many other disciplines: first understand how a system works in a pure setting and then generalize it step by step in order to transfer it to a more noisy real world scenario. In this paper we argue that in the case of neuroscience such an approach may well lead to misconceptions of fundamental principles of information processing in the brain. Our simulations of populations of neurons whose connectivities and properties are closely matched with biological data (cf. methods) show that variance in the synapses and neurons crucially changes the dynamics of the network. For example, the spikes in reliable, homogeneous networks tend to synchronize to a precision of a few milliseconds (and thus to a precision considerably higher than observed for behaving humans and animals). On the other hand, the same network with unreliable and heterogeneous synapses and neurons decreases these correlations (cf. Fig. 2 c,d). We also show that the amount of input activity needed in order to elicit activity is significantly smaller, and thus more energy efficient in a heterogeneous setup (Fig. 3 a–c). The same is true for the time it takes for a population of neurons to react to an external stimulation (Fig. 3d). The differences became even more distinctive when we used the output of one population as the input of another one as depicted in Figure 4.

Our results can also explain a discrepancy between experimental results and theoretical modeling/simulations present in current efforts to understand neuronal signal propagation. While some experiments show decorrelating effects [13], most simulations report increasing correlations [14]–[20]. And those that found propagation modes of stable or decreasing correlation needed to incorporate additional assumptions like *(i)* a high level of extrinsic noise [21], *(ii)* simultaneous convergence of multiple signals [20], [22], or *(iii)* unrealistically strong feed-forward synapses [19] (see also the section on related work). Here we show that decorrelation is actually possible without any of these additional assumptions. The existing discrepancy vanishes if we incorporate a realistic amount of heterogeneity in the network.

Another and perhaps more surprising result is that variability in the neuronal parameters guarantees stability. In order to study this we considered the dynamics of a recurrent network (see below) to distorted input. By this we mean the following. Two types of input that are both intensively studied are *(i)* ‘Poisson input’, where the input neurons spike *independently*, and *(ii)* synchronized input where the input arrives as a ‘flank’, meaning that the input neurons spike more or less *simultaneously*. Both modes have been observed in biological neural systems [23], and most likely both play important roles for information processing in the brain. While it is easy to generate independent Poisson spikes in simulations, it is still unclear how neuronal ensembles in the brain can generate Poisson spiking: wherever activity arises there are lateral/local connections and thus some dependencies between spike times. That is, even if a Poisson-like spiking occurs it will most likely be flawed with synchronization (as is well known from simulations [18]). In Fig. 2 c-f we show the effect of homogeneity/heterogeneity in such a scenario. We find that a heterogeneous network is not only immune to a certain amount of synchronized activity but can even remove or weaken them, while homogeneous networks increase them.

## Results and Discussion

We simulated populations of 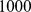 excitatory and 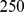 inhibitory conductance-based leaky integrate-and-fire neurons randomly interconnected with a connectivity of 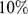 [24]. The neuronal and synaptic models come with many parameters, all of which we drew randomly for each neuron and synapse from a distribution based on physiological data in mammalian cortex (“heterogeneous network”, see methods). Moreover, we incorporated the fact that vesicle release of a synaptic site is unreliable [2]. In a second setup, we set all parameters to the mean values of their distributions and made the vesicle release 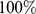 reliable (“homogeneous network”), normalizing the synaptic weights such that the expected postsynaptic current was the same in both setups. We studied the reaction of a population to perfect Poisson input and to Poisson input that was flawed by spontaneous synchronized activity in which all input neurons produced a spike within 5 ms. We control the amount of synchronization by a parameter 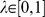 giving the fraction of spikes belonging to the flanks (so 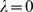 means Poisson spiking, and for 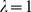 all spikes belong to flanks). The input was modeled by 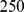 excitatory neurons, each projecting to randomly chosen 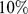 of the target population. The mean synaptic weights of lateral connections were chosen in such a way that excitation and inhibition were balanced [25]. That is, the total activity in the population shows input normalization [26] for a large range of input rates (Fig. 2 a). This does not mean that all neurons behave the same. For example, in the experiment from Figure 2 a with heterogeneous setup and 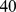 Hz input there more than 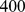 neurons firing with less than 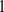 Hz, while 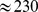 neurons fired with 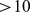 Hz, among them 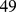 with 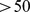 Hz. For other input rates and also for the homogeneous setup the corresponding numbers were very similar. Finally, the transmission delays of spikes were drawn randomly in both cases, as they depend on the geometry of the network and thus differ even if all neurons are identical (see methods).

We found that the global response of a population to Poisson input is very similar in both the heterogeneous and homogeneous case in terms of average output rate (Fig. 2 a). This was no surprise, as in a population of some thousand neurons the law of large numbers should diminish the effect of variations in the neuronal parameters. As a measure of synchrony, we computed the average cross-covariance (CC) of the binned spike trains of pairs of neurons (see methods for reasons), as done in the experimental paper [13]. The cross-correlation is a measure of the correlation between neuronal activity in a small time interval (“bin”) over several trials. A high CC indicates that for every bin neurons tend to fire jointly. A CC close to zero indicates that the precise spike time of one neuron does not have strong implications for the spikes times of other neurons in the same bin. The CC generally tends to be smaller for small bin sizes, since the number of spikes per bin is small in this case, and has been criticized for this reason (see [27] for a review). Therefore, we also compute the coefficient of variation (CV) of the interspike intervals of all spike events in each population. The CV is a measure for the irregularity of the interspike intervals, and does not suffer from the drawbacks of the CC. It is equal to 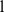 for Poisson spiking and greater than 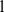 for correlated spike times. Note that the interspike intervals are *not* taken between spikes of the same neuron, but rather between any neurons of the network (see methods for the reasons).

We observed that the CV is slightly higher for the homogeneous network than in the heterogeneous case. In addition, the CV reacts to flawed Poisson input much stronger in the homogeneous case (Fig. 2 c–d). We also observed that this increase was not due to a single parameter, but had its cause rather in the interplay of many different sources of variance (Fig. 2 c). Moreover, different sources of variance add up in a non-linear way. For the CV, variance in the inhibitory neurons is especially important, since synchronous inhibition is able to diminish the output of the population drastically.

The picture became even more distinctive when we let the signal propagate along a feed-forward network of several such populations as depicted in Figure 4. It is known that synchrony tends to increase along such a feed-forward network (see section on related work). We studied a sequence of eight neuronal populations, with the excitatory cells in population 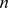 projecting to randomly chosen neurons in population 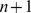 (see methods). In this way we could investigate how correlations evolve when a signal is propagated through several populations. While in the homogeneous network the CV increased as the signal propagated, the heterogeneous network remained close to being Poisson (CV approximately one) even in subsequent populations (Fig. 2e). Fig. 2f shows the evolution of the cross-correlation over several populations in a flawed Poisson setup (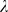 = 0.2). The cross-correlations of the homogenous networks (red) were larger than for the input (green) and increased from population to population. On the other hand, the heterogeneous network (blue) decorrelated the small disturbances in the input and then remained close to Poisson. Note also that this implies that in the heterogeneous case all eight populations behaved very similar, while the increasing synchronization in the homogeneous network led to significant changes in activity between several populations (data not shown).

At first sight it may seem that such a decorrelating network would perform poorly in processing input flanks like the ones appearing in gamma oscillations. However, recent work on purely excitatory networks has shown that varying spike thresholds can improve sensitivity to the input [28]. Our simulations show that this effect remains in decorrelating networks with balanced excitation and inhibition. When we tested the reaction of the networks to input flanks, heterogeneous networks showed not only a stronger response (Fig. 3 a–c), but also reacted faster (Fig. 3d). Moreover, the heterogeneous network was activated by fewer spikes, which makes it more energy efficient. Note that flanks in the input like those in Fig. 3 (that may be seen as carriers of information) as well as the synchronization in the flawed Poisson inputs are characterized by a high level of synchrony. The difference lies in the time scale: while for flawed Poisson input the spikes are synchronized up to a few milliseconds, the spikes of the input flank in Fig. 3 are scattered over 10–30 ms. Moreover, as the synchronization within Poisson input arises spontaneously and randomly [18], it does not carry meaningful information. The symmetry breaking properties of heterogeneous networks allow to distinguish between these two cases: they desynchronize the spontaneous synchronization and at the same time increase the effect of flanks as the carrier of information.

### Related Work

Although a lot of theoretical work has been invested into understanding neural signal propagation [10], [17]–[20], [29]–[34], no simulation could explain the decorrelation *in vivo* [13]. Most simulations reported increasing correlations [14]–[20], and those that found propagation modes of stable or decreasing correlation needed to incorporate assumptions incompatible with the experimental setup, like a high level of extrinsic noise [21], simultaneous convergence of multiple signals [20], [22], or unrealistically strong feed-forward synapses, up to 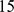-fold stronger than lateral synapses [19].

For other types of recurrent physical networks, a bit more is known. In particular, synchronization effects have been studied for networks of coupled oscillators [8], [35]–[38], which sometimes have been interpreted as networks of neuronal ensembles, or for networks of neurons whose membrane potential are directly coupled to each other [39]. Hansel and Mato [40] studied synchronization of networks of rate-based approximations to neurons [40]. Also networks of spiking neurons have been investigated for purely excitatory neurons [41], [42], or purely inhibitory neurons receiving constant input currents [42], [43]. Closest to our work are the embedded synfire chains considered in [44], and the Dale networks studied in [45]. Both are networks of spiking neurons, similar to the present paper. However, as both groups model the synaptic currents as delta peaks, and use uniform synaptic delays for all connections, the impact of synaptic time dynamics on synchronization were not investigated in these studies.

For the various networks, several properties have been shown to desynchronize the networks dynamics in simulations, including noise [39], [41], large system size [36], [46], and heterogeneity of synaptic strengths [8], [35], [40], [44] and connectivity [44], [45] and of other biophysical parameters [8], [37], [38], [42], [43].

Recently, Mejias and Longtin [28] have studied the effect of varying spike thresholds on synchrony in purely excitatory networks. They found that higher variance leads to *stronger* synchronization, an opposing effect to the one we observe. One important difference in our setup is that we use a balanced system of inhibition and excitation, cf. Figure 2 a. Mejias and Longtin observe that increasing variance in spike thresholds increases also the output rates. For single neurons it is well known [47] that synchronization rises with the output rate. In our balanced system the main effect of the variances is not a change of the output rate but instead a more subtle reaction of the population to varying input strengths, which in turn results in a more asynchronous behaviour.

In behaving animals and humans, the activity of clusters of neurons is oscillatory with frequencies of 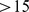 Hz [48]–[51], with most excitatory neurons firing highly irregular [52], named synchronous irregular (SI) state in [47]. Many experiments also reported strong spike count correlation on single cell level, for example of pyramidal cells in V1 with similar receptive fields [12], [53], [54]. Recently Ecker et al. [55] with permanently implanted tetrodes reported, in contrast to these results, that the correlations are in fact negligibly low when a high temporal resolution (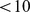 ms) is applied. They reasoned that previous, contradictory findings were an artifact of measurement [55] or analysis techniques [56], or were due to exceptionally high and polysynaptic input from LGN [53]. In light of their findings, Ecker et al. speculated about an active decorrelation process in the brain. Nevertheless, Cohen and Kohn [27] have in turn challenged the measurements and the interpretation of Ecker et al. such that a conclusive bottom-line can not yet been drawn. Our experiments may be viewed as a support of the speculations in [55].

Our results can be explained as follows. In accordance with the *law of large numbers*, the variance in the parameters plays only a negligible role if we study simple input-output systems without complicated dynamics. For the considered input ranges, our system is of this type, despite of the recurrent connections. In particular the input-response curve does not change much (Fig. 2a). However, variations in the neuronal parameters do have a *symmetry-breaking* effect that tremendously influences the local reactivity to changes in the input, cf. Fig. 2 c–d. Concretely, in a homogeneous network interneurons tend to react groupwise, thus easily over- or underreacting to pyramidal activity. When each interneuron has different integration properties, they can counterbalance the pyramidal activity more accurately. And as it is well known that functionality of the neuron system crucially depends on a careful and balanced interplay of excitation and inhibition [11], [12], such symmetry breaking effects make the system react in a more subtle and balanced way than in a homogeneous setup.

## Conclusion

While the benefits of a high variance are generally accepted in terms of the biodiversity of ecologic systems [57], the potential benefits for neural signal processing are still largely unexplored. We hope that a systematic exploration will be as fruitful as the study of noise in the field of stochastic resonance. In this paper we have undertaken a first step by showing that heterogeneity can enhance speed, responsiveness, and – counterintuitively – robustness of networks of spiking neurons. Our simulations show that various kinds of variance – from variances in neuronal parameters to unreliability of synapses – contribute to these effects. Quantifying the effects of the various parameters is hard, as the contributions do not seem to add up linearly, but depend on each other. We leave a more thorough study of these interdependencies to future work.

## Methods

### 1 Neuron Model

#### 1.1 Leaky Integrate and Fire Dynamics

All model neurons in our simulations are conductance-based leaky integrate-and-fire (LIF) [58], [59] neurons. Gerstner gives a thorough overview of LIF neurons [60]. All simulations were implemented within the NEST framework [61]. The dynamics of the current based LIF model are governed by the following differential equation:
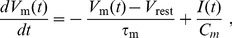
(1)where 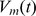 is the membrane voltage, 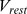 is the resting potential, 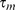 is the membrane time constant, and 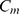 is the capacitance of the neuron's membrane. The post-synaptic current (PSC) 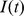 is determined by the time-dependent voltage and the time-dependent membrane conductance,



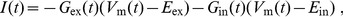
(2)where 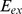 and 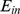 are the reversal potentials of excitatory ions and inhibitory (potassium) ions, respectively. We did not estimate conductances and capacitances separately, but only their quotients (cf. section 2.5). In case that synaptic input raises the membrane potential 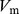 above the threshold potential 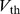, the cell elicits an action potential (spike) and all the neurons the cell projects to will receive conductance changes that express excitatory postsynaptic currents (EPSCs) if the projecting cell is excitatory, or inhibitory postsynaptic currents (IPSCs) if the projecting cell is an interneuron (see also below). After the generation of such a spike event, the neuron undergoes an absolute refractory period of 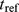 milliseconds (ms) in which it is incapable of generating further spikes. At the end of the absolute refractory period the cell's 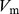 value is reset to 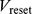. The conductance 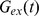 induced by the 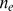 excitatory synapses is given by
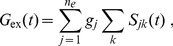
(3)where every presynaptic spike event contributes 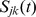 given by Equation (5) below, and 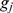 is the dimensionless strength of the connection, defined as the integrated conductance change induced at the soma divided by its capacitance 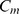. The conductance 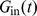 is analogously given by
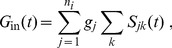
(4)where 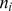 is the number of inhibitory synapses, and 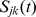 is given by Equation (6) below. The response curve 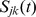 consists of 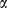-Amino-3-hydroxy-5-methyl-4-isoxazolepropionic acid (AMPA) and N-methyl-D-aspartat (NMDA) components for excitatory synapses,
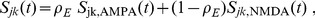
(5)where 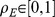 determines the ratio between AMPA- and NMDA-mediated conductance changes. For inhibitory synapses, the response curve is given by gamma-aminobutyric acid (GABA), so in this case we have



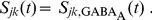
(6)AMPA and 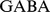 triggered conductance changes are modeled by (normalized) single exponentials [62]

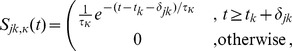
(7)where 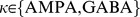, with typical decay time-constants of 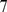 ms at pyramidal cells [62]–[64] and 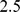 ms at interneurons [62]. Note that the integral over 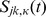 is normalized to 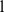. The term 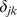 accounts for the axonal, synaptic, and dendritic delay of the synaptic connection. NMDA triggered currents are modeled by double exponentials [65]



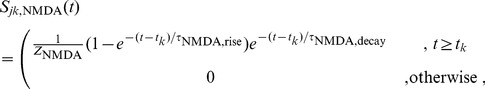
(8)with rise time-constant of 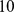 ms and decay time-constant of 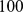 ms [64], [65]. The constant
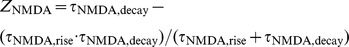
normalizes the integral over 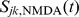 to 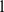.

### 2 Parameters

Table 1 and Table 2 provide the values for the parameters of neurons and synapses that we used in our simulations. Each parameter was drawn uniformly at random from an interval. Mean value, upper and lower bound of the interval are given in the tables. Note that the standard deviation of such a uniform distribution in 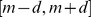 is given by 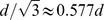.

**Table 1:** Neuronal parameters.

Excitatory Cells				Inhibitory Cells			
parameter	mean	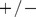	Unit	Parameter	Mean	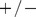	Unit
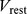	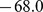	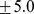	mV	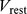	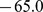	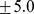	mV
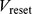	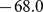	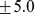	mV	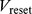	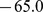	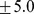	mV
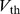	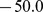	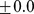	mV	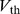	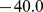	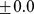	mV
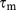	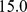	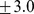	Ms	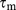	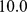	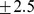	Ms
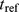	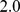	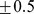	Ms	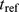	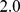	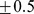	Ms
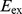	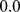	–	mV	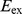	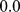	–	mV
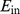	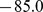	–	mV	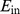	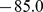	–	mV
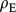	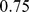	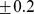	–	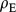	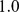	–	–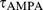	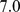	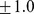	Ms	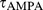	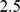	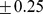	ms
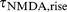	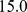	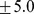	Ms	–			
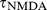	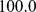	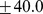	Ms	–			
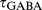	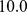	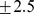	Ms	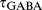	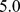	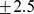	Ms

**Table 2:** Synaptic parameters. For a specifiction of 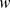 and the amplitude see section 2.5.

parameter	unit	A 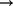 P	A 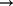 I	P 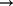 P	P 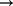 I	I 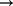 P	I 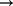 I
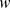	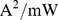	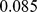 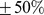	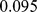 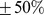	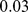 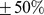	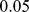 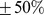	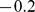 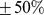	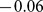 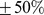
amplitude	mV	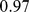 – 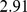	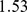 – 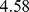	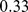 – 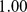	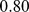 – 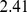	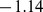 – 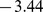	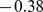 – 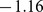
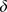	Ms	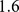 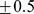	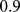 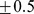	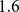 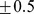	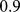 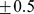	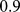 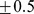	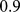 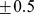
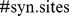	–	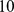	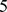	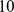	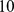	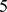	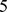
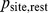	–	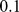	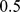	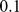	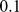	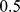	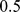

A: afferent pyramidal cells (neuron from previous population in the propagation chain, also used for connections between external input and first population). P: pyramidal cells. I: interneurons.

In the subsequent sections we provide an overview of experimental data on cortical pyramidal cells and cortical basket cells that justify the choice of these values. All the collected animal data was measured in cats, ferrets, and rodents.

#### 2.1 The Resting Potential

There is a long list of publications containing in vitro resting potential 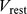 data in various animals [62], [66]–[81]. The in vitro spectrum of measured resting potentials in pyramidal cells ranges from 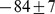 mV to 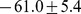 mV. We choose 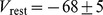 mV for pyramidal cells [68], [80]–[84] and 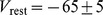 mV for interneurons [73]. This is also consistent with *in vivo* measurements [85], although there is again a wide range from 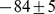 mV [86] to 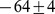 mV [87].

For inhibitory cells resting potential measured in vitro range from 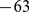 to 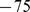 mV [62], [68], [71], [74]–[78], [88]–[91], with standard deviations from 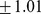 mV [77] to 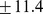 mV [88]. Measurements in vivo show slightly less negative resting potentials of 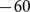
–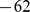 mV [92], possibly due to ongoing background activity. Unfortunately, the sample size (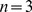) in [92] was too small to give reliable information on the standard deviation.

#### 2.2 The Threshold Potential

In vitro data for the threshold potential 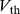 of individual excitatory or inhibitory neurons can be found in [72], [73], [79]–[81], [93]. Detailed in vivo data in rat prefrontal cortex can be found in Degenetais et al. [85]. For pyramidal cells they find parameter ranges from 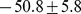 mV to 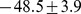 mV. In vitro data for basket cells can be found in [73] (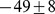 mV) or [93] (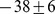 mV). Since varying the threshold potential has essentially the same effect as varying the resting potential, we restricted variations to the resting potential.

#### 2.3 Membrane Time Constant (Leakage)

A vast amount of data can be found for membrane time constants 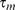 in various places in different animals for excitatory cells [62], [66]–[68], [70], [71], [76], [78], [80], [81], [94] and for inhibitory cells [62], [68], [71], [75], [76], [78], [89]–[91], [94], [95].

For pyramidal cells the cited studies offer values ranging from 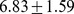 ms [70] to 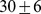 ms [62]. A majority of this data supports a membrane time constant of about 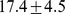 to 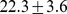 ms: [78], [94] in monkey, [66], [68] in guinea pig, and [76], [94] in rats.

For inhibitory cells values range from 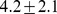 ms [91] to 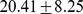 ms [75]. Most studies support mean values of 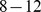 ms [62], [68], [71], [76], [78], [89], [90], [94] and standard deviations of 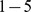 ms [62], [75], [76], [78], [89]–[91], [94], [95]

#### 2.4 Refractory Period

Measurements of the absolute refractory period are rare. Values of 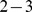 ms have been found *in vivo* in rat hippocampal pyramidal cells [96], and fast-spiking neurons tend to have similar refractory period as regular spiking neurons [97]. Note that the terminology of absolute and relative refractory period in the second references deviates from our terminology.

#### 2.5 Synaptic strength

In order not to estimate the synaptic capacitance and conductance separately, we rather fitted the total integral over the quotient 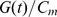, which we call *synaptic efficacy* 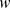. Intuitively, this parameter corresponds to the weight of the synapse. Table 2 shows the amplitude of the EPSP change that a single incoming spike evokes (for mean values of other synaptic and neuronal parameters) if the voltage is initially at the resting potential.

We did not derive the synaptic efficacies from literature, but we chose them in a way that inhibition in our populations balanced excitation (the gain in activity is close to 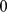 for a wide range of inputs). This was important for the homogeneous network to function properly, but not for the inhomogeneous. The latter one also showed a large stable operation range if the gain was positive (data not shown).

Although we did not directly fit efficacies to the literature, all used values are within the reported bounds.

Depending on the nature of the synaptic currents, 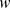 will be denoted with appropriate suffixes like 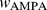, 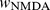, 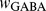, or 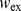 and 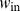 for total excitatory and inhibitory currents.

#### 2.6 AMPA mediated PSPs and PSCs

The AMPA time constant 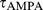 that we use is rarely estimated directly; more often the half-width of the EPSP is considered. Results for pyramidal cells are 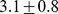 ms [63], 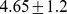 ms [98], 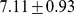 ms [62], 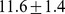 ms [99], and 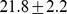 ms [99], for interneurons 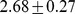 ms [62] and 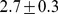 ms [99]. In general, the time constant and its variance are higher if only NMDA is blocked, but not other neurotransmitters like kainate [99]. We used the data from Karayannis et al. [62] since they measured both pyramidal cells and interneurons. Furthermore, the corresponding rise times and half-width of AMPA mediated PSPs in [62] match with other studies [76], [100], except that those studies find higher variances.

#### 2.7 NMDA mediated PSCs

We chose the values of 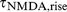 to match rise times between 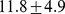 ms [64] and 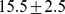 ms [65]. Decay rates 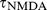 are reported in the range between 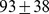 ms [64] and 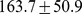 ms [65].

#### 2.8 AMPA/NMDA Ratio

We used data about the AMPA/NMDA ratio 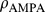 for synapses onto pyramidal neurons in [65], [101], [102], and in a review by Thomson et al. [103] for synapses onto inhibitory neurons. For pyramidal neurons 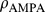 ranges from 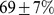 [101] to 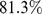 [65]. For excitatory connections onto inhibitory neurons the NMDA component seems to be much smaller or even absent [103]. Therefore we did not incorporate NMDA components in the latter case.

#### 2.9 GABA mediated PSCs

As for the EPSC, there is only sparse data on the decay time constant 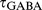 of inhibitory postsynaptic currents (IPSCs). For pyramidal cells, Wang et al. [75] find 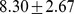 ms, for fast spiking cells Tamas et al. [88] report 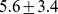 ms. This is consistent with the finding of Thomson et al. [104] that the IPSP rise time is about twice as large in pyramidal cells compared two interneurons.

#### 2.10 Synaptic Sites and Reliability

There are typically 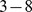 synaptic contacts between any two pyramidal cells in layer 5 [101], [105], [106], each of them having potentially more than one vesicle release site. The transmission probability of the total dendritic tree within one layer is 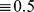 [107], while it is 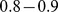 for projections between layers. Unfortunately, estimates on quantal count and quantal release probability are highly contradictory (see [108] for an overview). Therefore, we decided to assume 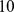 vesicle release sites [106] with a resting release probability of 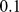 for all glutamatergic synapses, regardless of the innervating cell type. For GABAergic synapses, we assumed 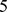 vesicle release sites with a resting release probability of 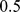 to account for the low transmission failure rate of basket cells [71].

#### 2.11 Latencies

The time constant 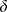 accounts for the time lag between generation of an action potential in a presynaptic neuron and the arrival of the EPSC at the postsynaptic soma. Hence it includes axonal, synaptic and dendritic delay. For close-by neurons (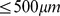), connections between pyramidal cells have a delay between 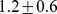 ms and 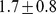 ms [82], [109], [110]. Connections to and from basket cells have been reported to be faster: between 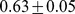 ms and 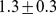 ms from pyramidal cells to basket cells [74], [75], [109]; between 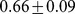 ms and 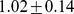 ms from pyramidal cells to basket cells [75], [77], [90], [94]; and between 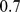 ms and 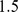 ms from basket cells to basket cells (no standard deviation given) [109]. In all the latter cases, a majority of measurements supports a value of roughly 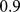 ms [75], [77], [94], [109]

### 3 Feed-Forward Network

#### 3.1 Populations

The network consisted of a feed forward chain of up to 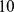 populations. Each population consisted of 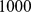 pyramidal cells and 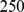 interneurons. Within each population, each neuron projected to each other neuron with probability 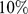 [111], as depicted in Figure 4. The probability was the same for pyramidal cells and interneurons. Moreover, each pyramidal cell in population 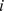 projected to each (excitatory or inhibitory) neuron in population 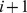 with probability 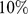. Interneurons did not project to other populations, as cortical basket cells do rarely project into other layers. [112].

#### 3.2 Input

There are 250 excitatory input neurons giving input to the network. For Fig. 2 (f,g) the input goes only to the first population. As for the connections between population, each input neuron projects to each neuron in the next population with probability 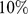. For Figure 2, each input neuron emits a Poisson spike train of fixed rate. For Figure 3, a random subset of the input neurons each emits a spike at a randomly chosen time in some small, predefined interval. We speak of a “flank” of input spikes in this case.

### 4 Measures of synchrony

The *coefficient of variation* (CV) of the spike time intervals was computed as follows. Let 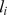 be the time between the 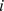
th and the 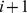
st pyramidal spike (not necessarily of the same neuron). Then we computed the mean 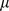, the standard variation 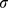, and the coefficient of variation CV as
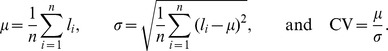



Many experimental and theoretical papers consider the CV of the spike time intervals of a single neuron (between two spikes of this specific neuron), and possibly average this value over many neurons. This individual CV serves other purposes, and should not be confused with the population-CV that we compute. In particular, the individual CVs can not serve as measures of synchrony: Consider a perfectly synchronous system of neurons which all spike at exactly the same times, but these times are random. Then each individual neuron will have a CV close to 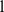. On the contrary, the population-CV of such a system will be extremely high: while most spike time intervals are close to 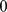, there are some (comparably) extremely long time intervals in which the complete system is silent.

The *cross-correlation* (CC) of the binned spike times was computed as follows. We repeated the experiment 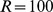 times. Then we binned the time of the experiments with some bin size 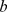 (for the value of 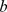, see figures). For each neuron 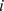 and each trial 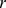 we counted the number of spikes 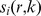 of neuron 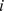 that occurred in the 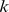
th bin in trial 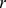. We computed the average number of spikes 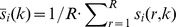 of neuron 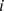 in bin 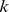. Then the cross-correlation between two neurons 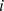 and 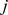 in the 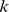
th bin was calculated as
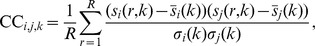
where 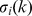 and 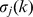 are the standard deviation of 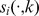 and 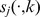, respectively. Finally, the cross-correlation CC was computed as the mean of 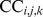, taken over all neurons 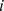 and 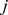 and all bins 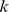.

## References

[1] Mielke N, Marquart T, Wu N, Wu N, Kessenich J, et al.. (2007) Bit error rate in NAND Flash memories. Audio, Transactions of the IRE Professional Group on: 9–19.

[2] Stevens CF, Wang Y (1994) Changes in reliability of synaptic function as a mechanism for plasticity. Nature 371: 704–707. 7935816 10.1038/371704a0

[3] Stein RB, Gossen ER, Jones KE (2005) Neuronal variability: noise or part of the signal? Nature Reviews Neuroscience 6: 389–397. 15861181 10.1038/nrn1668

[4] Urban N, Tripathy S (2012) Neuroscience: Circuits drive cell diversity. Nature 488: 289–290. 22895331 10.1038/488289a

[5] Douglas JK, Wilkens L, Pantazelou E, Moss F (1993) Noise Enhancement of Information Transfer in Crayfish Mechanoreceptors by Stochastic Resonance. Nature 365: 337+. 10.1038/365337a0 8377824

[6] Wiesenfeld K, Moss F (1995) Stochastic resonance and the benefits of noise: from ice ages to crayfish and SQUIDs. Nature 373: 33–36. 7800036 10.1038/373033a0

[7] Moss F (2004) Stochastic resonance and sensory information processing: a tutorial and review of application. Clinical Neurophysiology 115: 267–281. 14744566 10.1016/j.clinph.2003.09.014

[8] Denker M, Timme M, Diesmann M, Wolf F, Geisel T (2004) Breaking synchrony by heterogeneity in complex networks. Physical review letters 92: 074103. 14995855 10.1103/PhysRevLett.92.074103

[9] Gerstner W, Kreiter AK, Markram H, Herz AV (1997) Neural codes: firing rates and beyond. Proceedings of the National Academy of Sciences of the United States of America 94: 12740–12741. 9398065 10.1073/pnas.94.24.12740 PMC34168

[10] Kumar A, Rotter S, Aertsen A (2010) Spiking activity propagation in neuronal networks: reconciling different perspectives on neural coding. Nat Rev Neurosci 11: 615–27. 20725095 10.1038/nrn2886

[11] Lampl I M Okun (2009) Balance of excitation and inhibition. Scholarpedia 4: 7467.

[12] Okun M, Lampl I (2008) Instantaneous correlation of excitation and inhibition during ongoing and sensory-evoked activities. Nature Neuroscience 11: 535–7. 18376400 10.1038/nn.2105

[13] Ranganathan GN, Koester HJ (2011) Correlations decrease with propagation of spiking activity in the mouse barrel cortex. Frontiers in Neural Circuits. 10.3389/fncir.2011.00008 PMC3099309 21629764

[14] Marsálek P, Koch C, Maunsell J (1997) On the relationship between synaptic input and spike output jitter in individual neurons. Proceedings of the National Academy of Sciences of the United States of America 94: 735–40. 9012854 10.1073/pnas.94.2.735 PMC19583

[15] Burkitt AN, Clark GM (1999) Analysis of integrate-and-fire neurons: synchronization of synaptic input and spike output. Neural Computation 11: 871–901. 10226187 10.1162/089976699300016485

[16] Diesmann M, Gewaltig MO, Aertsen A (1999) Stable propagation of synchronous spiking in cortical neural networks. Nature 402: 529–33. 10591212 10.1038/990101

[17] Mehring C, Hehl U, Kubo M, Diesmann M, Aertsen A (2003) Activity dynamics and propagation of synchronous spiking in locally connected random networks. Biol Cybern 88: 395–408. 12750902 10.1007/s00422-002-0384-4

[18] Reyes AD (2003) Synchrony-dependent propagation of firing rate in iteratively constructed networks in vitro. Nature Neuroscience 6: 593–9. 12730700 10.1038/nn1056

[19] Vogels T, Abbott L (2005) Signal propagation and logic gating in networks of integrate-and-fire neurons. The Journal of Neuroscience 25: 10786. 16291952 10.1523/JNEUROSCI.3508-05.2005 PMC6725859

[20] Kumar A, Rotter S, Aertsen A (2008) Conditions for propagating synchronous spiking and asynchronous firing rates in a cortical network model. Journal of Neuroscience 28: 5268–5280. 18480283 10.1523/JNEUROSCI.2542-07.2008 PMC6670637

[21] van Rossum MCW, Turrigiano GG, Nelson SB (2002) Fast propagation of firing rates through layered networks of noisy neurons. J Neurosci 22: 1956–66. 11880526 10.1523/JNEUROSCI.22-05-01956.2002 PMC6758872

[22] Vogels T, Abbott L (2009) Gating multiple signals through detailed balance of excitation and inhibition in spiking networks. Nature Neuroscience 12: 483–491. 19305402 10.1038/nn.2276 PMC2693069

[23] Uhlhaas PJ, Pipa G, Lima B, Melloni L, Neuenschwander S, et al.. (2009) Neural Synchrony in Cortical Networks: History, Concept and Current Status. Frontiers in Integrative Neuroscience 3. 10.3389/neuro.07.017.2009 PMC2723047 19668703

[24] Kalisman N, Silberberg G, Markram H (2005) The neocortical microcircuit as a tabula rasa. Proceedings of the National Academy of Sciences of the United States of America 102: 880–5. 15630093 10.1073/pnas.0407088102 PMC545526

[25] Isaacson JS, Scanziani M (2011) How Inhibition Shapes Cortical Activity. Neuron 72: 231–243. 22017986 10.1016/j.neuron.2011.09.027 PMC3236361

[26] Pouille F, Marin-Burgin A, Adesnik H, Atallah B, Scanziani M (2009) Input normalization by global feedforward inhibition expands cortical dynamic range. Nature Neuroscience 12: 1577–85. 19881502 10.1038/nn.2441

[27] Cohen MR, Kohn A (2011) Measuring and interpreting neuronal correlations. Nature Neuroscience 14: 811–819. 21709677 10.1038/nn.2842 PMC3586814

[28] Mejias JF, Longtin A (2012) Optimal heterogeneity for coding in spiking neural networks. Physical Review Letters 108: 228102. 23003656 10.1103/PhysRevLett.108.228102

[29] Destexhe A, Contreras D (2006) Neuronal computations with stochastic network states. Science 314: 85–90. 17023650 10.1126/science.1127241

[30] Kremkow J, Perrinet LU, Masson GS, Aertsen A (2010) Functional consequences of correlated excitatory and inhibitory conductances in cortical networks. Journal of computational neuroscience 28: 579–94. 20490645 10.1007/s10827-010-0240-9

[31] Kremkow J, Aertsen A, Kumar A (2010) Gating of signal propagation in spiking neural networks by balanced and correlated excitation and inhibition. The Journal of Neuroscience 30: 15760. 21106815 10.1523/JNEUROSCI.3874-10.2010 PMC6633769

[32] Motter A (2010) Nonlinear dynamics: Spontaneous synchrony breaking. Nature Physics 6: 164–165.

[33] Aviel Y, Mehring C, Abeles M, Horn D (2003) On embedding synfire chains in a balanced network. Neural Comput 15: 1321–40. 12816575 10.1162/089976603321780290

[34] Tetzlaff T, Buschermöhle M, Geisel T, Diesmann M (2003) The spread of rate and correlation in stationary cortical networks. Neurocomputing 52: 949–954.

[35] Börgers C, Kopell N (2003) Synchronization in networks of excitatory and inhibitory neurons with sparse, random connectivity. Neural Comput 15: 509–38. 12620157 10.1162/089976603321192059

[36] Golomb D, Hansel D (2000) The number of synaptic inputs and the synchrony of large, sparse neuronal networks. Neural Comput 12: 1095–139. 10905810 10.1162/089976600300015529

[37] Golomb D, Rinzel J (1993) Dynamics of globally coupled inhibitory neurons with heterogeneity. Phys Rev E Stat Phys Plasmas Fluids Relat Interdiscip Topics 48: 4810–4814. 9961165 10.1103/physreve.48.4810

[38] Tsodyks M, Mitkov I, Sompolinsky H (1993) Pattern of synchrony in inhomogeneous networks of oscillators with pulse interactions. Physical review letters 71: 1280–1283. 10055496 10.1103/PhysRevLett.71.1280

[39] Ostojic S, Brunel N, Hakim V (2009) Synchronization properties of networks of electrically coupled neurons in the presence of noise and heterogeneities. Journal of computational neuroscience 26: 369–392. 19034642 10.1007/s10827-008-0117-3

[40] Hansel D, Mato G (2003) Asynchronous states and the emergence of synchrony in large networks of interacting excitatory and inhibitory neurons. Neural Computation 15: 1–56. 12590818 10.1162/089976603321043685

[41] Hansel D, Mato G, Meunier C (1995) Synchrony in excitatory neural networks. Neural Comput 7: 307–37. 8974733 10.1162/neco.1995.7.2.307

[42] Neltner L, Hansel D, Mato G, Meunier C (2000) Synchrony in heterogeneous networks of spiking neurons. Neural Comput 12: 1607–41. 10935920 10.1162/089976600300015286

[43] White J, Chow C, Rit J, Soto-Treviño C, Kopell N (1998) Synchronization and oscillatory dynamics in heterogeneous, mutually inhibited neurons. Journal of computational neuroscience 5: 5–16. 9580271 10.1023/a:1008841325921

[44] Tetzlaff T, Morrison A, Timme M, Diesmann M (2004) Heterogeneity breaks global synchrony in large networks. Neurobiology 8: 258701–258701.

[45] Kriener B, Tetzlaff T, Aertsen A, Diesmann M, Rotter S (2008) Correlations and population dynamics in cortical networks. Neural Computation 20: 2185–2226. 18439141 10.1162/neco.2008.02-07-474

[46] Tetzlaff T, Morrison A, Geisel T, Diesmann M (2004) Consequences of realistic network size on the stability of embedded synfire chains. Neurocomputing 58: 117–121.

[47] Brunel N (2000) Dynamics of sparsely connected networks of excitatory and inhibitory spiking neurons. Journal of computational neuroscience. 10.1023/a:1008925309027 10809012

[48] Steriade M, Contreras D, Amzica F, Timofeev I (1996) Synchronization of fast (30–40 hz) spontaneous oscillations in intrathalamic and thalamocortical networks. J Neurosci 16: 2788–808. 8786454 10.1523/JNEUROSCI.16-08-02788.1996 PMC6578775

[49] Steriade M, Amzica F, Contreras D (1996) Synchronization of fast (30–40 hz) spontaneous cortical rhythms during brain activation. J Neurosci 16: 392–417. 8613806 10.1523/JNEUROSCI.16-01-00392.1996 PMC6578724

[50] Steriade M (2000) Corticothalamic resonance, states of vigilance and mentation. Neuroscience 101: 243–76. 11074149 10.1016/s0306-4522(00)00353-5

[51] Khazipov R, Luhmann HJ (2006) Early patterns of electrical activity in the developing cerebral cortex of humans and rodents. Trends Neurosci 29: 414–8. 16713634 10.1016/j.tins.2006.05.007

[52] Softky WR, Koch C (1993) The highly irregular firing of cortical cells is inconsistent with temporal integration of random epsps. J Neurosci 13: 334–50. 8423479 10.1523/JNEUROSCI.13-01-00334.1993 PMC6576320

[53] Lampl I, Reichova I, Ferster D (1999) Synchronous membrane potential fluctuations in neurons of the cat visual cortex. Neuron 22: 361–374. 10069341 10.1016/s0896-6273(00)81096-x

[54] Smith MA, Kohn A (2008) Spatial and temporal scales of neuronal correlation in primary visual cortex. J Neurosci 28: 12591–603. 19036953 10.1523/JNEUROSCI.2929-08.2008 PMC2656500

[55] Ecker AS, Berens P, Keliris GA, Bethge M, Logothetis NK, et al (2010) Decorrelated neuronal firing in cortical microcircuits. Science 327: 584–7. 20110506 10.1126/science.1179867

[56] Tetzlaff T, Rotter S, Stark E, Abeles M, Aertsen A, et al (2008) Dependence of neuronal correlations on filter characteristics and marginal spike train statistics. Neural Comput 20: 2133–84. 18439140 10.1162/neco.2008.05-07-525

[57] Chivian E, Bernstein A, of the Convention on Biological Diversity S, Programme UND, Programme UNE, et al. (2008) Sustaining Life: How Human Health Depends on Biodiversity. Oxford University Press.

[58] Tuckwell HC (1988) Introduction to theoretical neurobiology. Cambridge studies in mathematical biology, 8. Cambridge University Press.

[59] Dayan P, Abbott LF (2001) Theoretical Neuroscience: Computational and Mathematical Modeling of Neural Systems. The MIT Press, 1st edition.

[60] Gerstner W, Kistler WM (2002) Spiking Neuron Models: Single Neurons, Populations, Plasticity. Cambridge University Press.

[61] Diesmann M, Gewaltig M (2002) Nest: An environment for neural systems simulations. Gesellschaft für wissenschaftliche Datenverarbeitung mbH Göttingen: 43.

[62] Karayannis T, Huerta-Ocampo I, Capogna M (2007) Gabaergic and pyramidal neurons of deep cortical layers directly receive and differently integrate callosal input. Cereb Cortex 17: 1213–26. 16829551 10.1093/cercor/bhl035

[63] Hestrin S, Nicoll RA, Perkel DJ, Sah P (1990) Analysis of excitatory synaptic action in pyramidal cells using whole-cell recording from rat hippocampal slices. J Physiol (Lond) 422: 203–25. 1972190 10.1113/jphysiol.1990.sp017980 PMC1190128

[64] Hestrin S, Sah P, Nicoll RA (1990) Mechanisms generating the time course of dual component excitatory synaptic currents recorded in hippocampal slices. Neuron 5: 247–53. 1976014 10.1016/0896-6273(90)90162-9

[65] Watt AJ, van Rossum MC, MacLeod KM, Nelson SB, Turrigiano GG (2000) Activity coregulates quantal ampa and nmda currents at neocortical synapses. Neuron 26: 659–70. 10896161 10.1016/s0896-6273(00)81202-7

[66] Scholfield CN (1978) Electrical properties of neurones in the olfactory cortex slice in vitro. J Physiol (Lond) 275: 535–46. 633153 10.1113/jphysiol.1978.sp012206 PMC1282561

[67] Connors BW, Gutnick MJ, Prince DA (1982) Electrophysiological properties of neocortical neurons in vitro. Journal of Neurophysiology 48: 1302–20. 6296328 10.1152/jn.1982.48.6.1302

[68] McCormick DA, Connors BW, Lighthall JW, Prince DA (1985) Comparative electrophysiology of pyramidal and sparsely spiny stellate neurons of the neocortex. Journal of Neurophysiology 54: 782–806. 2999347 10.1152/jn.1985.54.4.782

[69] Deisz RA, Prince DA (1989) Frequency-dependent depression of inhibition in guinea-pig neocortex in vitro by gabab receptor feed-back on gaba release. J Physiol (Lond) 412: 513–41. 2557431 10.1113/jphysiol.1989.sp017629 PMC1190589

[70] Thomson AM, West DC (1993) Fluctuations in pyramid-pyramid excitatory postsynaptic potentials modified by presynaptic firing pattern and postsynaptic membrane potential using paired intracellular recordings in rat neocortex. Neuroscience 54: 329–46. 8336828 10.1016/0306-4522(93)90256-f

[71] Buhl EH, Cobb SR, Halasy K, Somogyi P (1995) Properties of unitary ipsps evoked by anatomically identified basket cells in the rat hippocampus. Eur J Neurosci 7: 1989–2004. 8528474 10.1111/j.1460-9568.1995.tb00721.x

[72] Stuart G, Sakmann B (1995) Amplification of epsps by axosomatic sodium channels in neocortical pyramidal neurons. Neuron 15: 1065–76. 7576650 10.1016/0896-6273(95)90095-0

[73] Fricker D, Verheugen JA, Miles R (1999) Cell-attached measurements of the firing threshold of rat hippocampal neurones. J Physiol (Lond) 517 (Pt 3): 791–804. 10.1111/j.1469-7793.1999.0791s.x PMC2269376 10358119

[74] Galarreta M, Hestrin S (2001) Spike transmission and synchrony detection in networks of gabaergic interneurons. Science 292: 2295–9. 11423653 10.1126/science.1061395

[75] Wang Y, Gupta A, Toledo-Rodriguez M, Wu CZ, Markram H (2002) Anatomical, physiological, molecular and circuit properties of nest basket cells in the developing somatosensory cortex. Cereb Cortex 12: 395–410. 11884355 10.1093/cercor/12.4.395

[76] Beierlein M, Gibson JR, Connors BW (2003) Two dynamically distinct inhibitory networks in layer 4 of the neocortex. J Neurophysiol 90: 2987–3000. 12815025 10.1152/jn.00283.2003

[77] Gao WJ, Wang Y, Goldman-Rakic PS (2003) Dopamine modulation of perisomatic and peridendritic inhibition in prefrontal cortex. J Neurosci 23: 1622–30. 12629166 10.1523/JNEUROSCI.23-05-01622.2003 PMC6741986

[78] González-Burgos G, Krimer LS, Urban NN, Barrionuevo G, Lewis DA (2004) Synaptic efficacy during repetitive activation of excitatory inputs in primate dorsolateral prefrontal cortex. Cereb Cortex 14: 530–42. 15054069 10.1093/cercor/bhh015

[79] Rodriguez-Molina VM, Aertsen A, Heck DH (2007) Spike timing and reliability in cortical pyramidal neurons: Effects of epsc kinetics, input synchronization and background noise on spike timing. PLoS ONE 2: e319. 17389910 10.1371/journal.pone.0000319 PMC1828624

[80] Kumar P, Ohana O (2008) Inter- and intralaminar subcircuits of excitatory and inhibitory neurons in layer 6a of the rat barrel cortex. Journal of Neurophysiology 100: 1909–22. 18650305 10.1152/jn.90684.2008

[81] Ledergerber D, Larkum ME (2010) Properties of layer 6 pyramidal neuron apical dendrites. J Neurosci 30: 13031–44. 20881121 10.1523/JNEUROSCI.2254-10.2010 PMC6633503

[82] Mason A, Nicoll A, Stratford K (1990) Synaptic transmission between individual pyramidal neurons of the rat visual cortex in vitro. The Journal of Neuroscience 11: 72–84. 10.1523/JNEUROSCI.11-01-00072.1991 PMC6575189 1846012

[83] Mason A, Larkman A (1990) Correlations between morphology and electrophysiology of pyramidal neurons in slices of rat visual cortex. II. Electrophysiology. The Journal of Neuroscience 10: 1415–1428. 2332788 10.1523/JNEUROSCI.10-05-01415.1990 PMC6570062

[84] Holmgren C, Harkany T, Svennenfors B, Zilberter Y (2003) Pyramidal cell communication within local networks in layer 2/3 of rat neocortex. J Physiol (Lond) 551: 139–53. 12813147 10.1113/jphysiol.2003.044784 PMC2343144

[85] Dégenètais E, Thierry AM, Glowinski J, Gioanni Y (2002) Electrophysiological properties of pyramidal neurons in the rat prefrontal cortex: an in vivo intracellular recording study. Cereb Cortex 12: 1–16. 11734528 10.1093/cercor/12.1.1

[86] Margrie TW, Brecht M, Sakmann B (2002) In vivo, low-resistance, whole-cell recordings from neurons in the anaesthetized and awake mammalian brain. Pugers Arch 444: 491–8. 10.1007/s00424-002-0831-z 12136268

[87] Baranyi A, Szente MB, Woody CD (1993) Electrophysiological characterization of different types of neurons recorded in vivo in the motor cortex of the cat. ii. membrane parameters, action potentials, current-induced voltage responses and electrotonic structures. Journal of Neurophysiology 69: 1865–79. 8350127 10.1152/jn.1993.69.6.1865

[88] Tamas G, Somogyi P, Buhl E (1998) Differentially interconnected networks of GABAergic interneurons in the visual cortex of the cat. The Journal of Neuroscience 18: 4255–4270. 9592103 10.1523/JNEUROSCI.18-11-04255.1998 PMC6792813

[89] Buhl EH, Szilágyi T, Halasy K, Somogyi P (1996) Physiological properties of anatomically identified basket and bistratified cells in the CA1 area of the rat hippocampus in vitro. Hippocampus 6: 294–305. 8841828 10.1002/(SICI)1098-1063(1996)6:3<294::AID-HIPO7>3.0.CO;2-N

[90] Gonzalez-Burgos G (2004) Functional Properties of Fast Spiking Interneurons and Their Synaptic Connections With Pyramidal Cells in Primate Dorsolateral Prefrontal Cortex. Journal of Neurophysiology 93: 942–953. 15385591 10.1152/jn.00787.2004

[91] Thomson AMA, Deuchars JJ, West DCD (1993) Single axon excitatory postsynaptic potentials in neocortical interneurons exhibit pronounced paired pulse facilitation. Neuroscience 54: 347–360. 8336829 10.1016/0306-4522(93)90257-g

[92] Ylinen A, Soltész I, Bragin A, Penttonen M, Sik A, et al (1995) Intracellular correlates of hippocampal theta rhythm in identified pyramidal cells, granule cells, and basket cells. Hippocampus 5: 78–90. 7787949 10.1002/hipo.450050110

[93] Fricker D, Miles R (2000) Epsp amplification and the precision of spike timing in hippocampal neurons. Neuron 28: 559–69. 11144364 10.1016/s0896-6273(00)00133-1

[94] Povysheva NV, Gonzalez-Burgos G, Zaitsev AV, Kröner S, Barrionuevo G, et al (2006) Properties of excitatory synaptic responses in fast-spiking interneurons and pyramidal cells from monkey and rat prefrontal cortex. Cereb Cortex 16: 541–52. 16033926 10.1093/cercor/bhj002

[95] Pawelzik H, Hughes DI, Thomson AM (2002) Modulation of inhibitory autapses and synapses on rat CA1 interneurones by GABAa receptor ligands. The Journal of Physiology 546: 701–716. 10.1113/jphysiol.2002.035121 PMC2342589 12562998

[96] Hirase H, Czurkó A, Csicsvari J, Buzsáki G (1999) Firing rate and theta-phase coding by hippocampal pyramidal neurons during ‘space clamping’. Eur J Neurosci 11: 4373–4380. 10594664 10.1046/j.1460-9568.1999.00853.x

[97] Chen N, Chen S, Wu Y, Wang J (2006) The refractory periods and threshold potentials of sequential spikes measured by whole-cell recording. Biochemical and Biophysical Research Communications 340: 151–157. 16343428 10.1016/j.bbrc.2005.11.170

[98] Diamond J, Jahr C (1995) Asynchronous Release of Synaptic Vesicles Determines the Time-Course of the Ampa Receptor-Mediated Epsc. Neuron 15: 1097–1107. 7576653 10.1016/0896-6273(95)90098-5

[99] Cossart R, Epsztein J, Tyzio R, Becq H, Hirsch J, et al (2002) Quantal release of glutamate generates pure kainate and mixed AMPA/kainate EPSCs in hippocampal neurons. Neuron 35: 147–159. 12123615 10.1016/s0896-6273(02)00753-5

[100] Tarczy-Hornoch K, Martin K, Stratford K, Jack J (1999) Intracortical excitation of spiny neurons in layer 4 of cat striate cortex in vitro. Cereb Cortex 9: 833–843. 10601002 10.1093/cercor/9.8.833

[101] Markram H, Lübke J, Frotscher M, Roth A, Sakmann B (1997) Physiology and anatomy of synaptic connections between thick tufted pyramidal neurones in the developing rat neocortex. J Physiol (Lond) 500 (Pt 2): 409–40. 10.1113/jphysiol.1997.sp022031 PMC1159394 9147328

[102] Myme C, Sugino K, Turrigiano G, Nelson S (2003) The nmda-to-ampa ratio at synapses onto layer 2/3 pyramidal neurons is conserved across prefrontal and visual cortices. J Neurophysiol 90: 771. 12672778 10.1152/jn.00070.2003

[103] Thomson AM, Deuchars J (1994) Temporal and spatial properties of local circuits in neocortex. Trends Neurosci 17: 119–26. 7515528 10.1016/0166-2236(94)90121-x

[104] Thomson A, West D, Wang Y, Bannister A (2002) Synaptic connections and small circuits involving excitatory and inhibitory neurons in layers 2–5 of adult rat and cat neocortex: Triple intracellular recordings and biocytin labelling in vitro. Cereb Cortex 12: 936–953. 12183393 10.1093/cercor/12.9.936

[105] Feldmeyer D, Sakmann B (2000) Synaptic efficacy and reliability of excitatory connections between the principal neurones of the input (layer 4) and output layer (layer 5) of the neocortex. The Journal of Physiology 525: 31–39. 10811722 10.1111/j.1469-7793.2000.00031.x PMC2269927

[106] Branco T, Staras K, Darcy KJ, Goda Y (2008) Local dendritic activity sets release probability at hippocampal synapses. Neuron 59: 475–485. 18701072 10.1016/j.neuron.2008.07.006 PMC6390949

[107] Gil Z, Connors BW, Amitai Y (1999) Efficacy of thalamocortical and intracortical synaptic connections: quanta, innervation, and reliability. Neuron 23: 385–397. 10399943 10.1016/s0896-6273(00)80788-6

[108] Branco T, Staras K (2009) The probability of neurotransmitter release: variability and feedback control at single synapses. Nature Reviews Neuroscience 10: 373–383. 19377502 10.1038/nrn2634

[109] Thomson AM, West DC, Wang Y, Bannister AP (2002) Synaptic connections and small circuits involving excitatory and inhibitory neurons in layers 2–5 of adult rat and cat neocortex: triple intracellular recordings and biocytin labelling in vitro. Cereb Cortex 12: 936–53. 12183393 10.1093/cercor/12.9.936

[110] Thomson AMA, Deuchars JJ, West DCD (1993) Large, deep layer pyramid-pyramid single axon EPSPs in slices of rat motor cortex display paired pulse and frequency-dependent depression, mediated presynaptically and self-facilitation, mediated postsynaptically. Journal of Neurophysiology 70: 2354–2369. 8120587 10.1152/jn.1993.70.6.2354

[111] Perin R, Berger TK, Markram H (2011) A synaptic organizing principle for cortical neuronal groups. Proceedings of the National Academy of Sciences of the United States of America 108: 5419–5424. 21383177 10.1073/pnas.1016051108 PMC3069183

[112] Binzegger T, Douglas RJ, Martin KAC (2009) Topology and dynamics of the canonical circuit of cat V1. Neural Networks 22: 1071–1078. 19632814 10.1016/j.neunet.2009.07.011

